# The genetic and environmental aetiology of spatial, mathematics and general anxiety

**DOI:** 10.1038/srep42218

**Published:** 2017-02-21

**Authors:** Margherita Malanchini, Kaili Rimfeld, Nicholas G. Shakeshaft, Maja Rodic, Kerry Schofield, Saskia Selzam, Philip S. Dale, Stephen A. Petrill, Yulia Kovas

**Affiliations:** 1 MRC Social, Genetic and Developmental Psychiatry Centre, King’s College London, UK; 2 Tomsk State University, Russia; 3 Department of Psychology, University of Sussex, UK; 4 Department of Speech and Hearing Science, University of New Mexico, Albuquerque, NM, USA; 5 Department of Psychology, The Ohio State University, Columbus, OH, USA; 6 Department of Psychology, Goldsmiths University of London, UK

## Abstract

Individuals differ in their level of general anxiety as well as in their level of anxiety towards specific activities, such as mathematics and spatial tasks. Both specific anxieties correlate moderately with general anxiety, but the aetiology of their association remains unexplored. Moreover, the factor structure of spatial anxiety is to date unknown. The present study investigated the factor structure of spatial anxiety, its aetiology, and the origins of its association with general and mathematics anxiety in a sample of 1,464 19-21-year-old twin pairs from the UK representative Twins Early Development Study. Participants reported their general, mathematics and spatial anxiety as part of an online battery of tests. We found that spatial anxiety is a multifactorial construct, including two components: navigation anxiety and rotation/visualization anxiety. All anxiety measures were moderately heritable (30% to 41%), and non-shared environmental factors explained the remaining variance. Multivariate genetic analysis showed that, although some genetic and environmental factors contributed to all anxiety measures, a substantial portion of genetic and non-shared environmental influences were specific to each anxiety construct. This suggests that anxiety is a multifactorial construct phenotypically and aetiologically, highlighting the importance of studying anxiety within specific contexts.

The negative relationship between general anxiety and cognitive and academic performance is now well documented^1^. High levels of anxiety have been associated with a wide range of negative educational outcomes, including poor academic achievement, early school leaving and failure to succeed in higher education^2^. A large literature review^3^ and a meta-analysis^4^ have observed moderate effects in the negative associations between general anxiety and academic performance (average *r* = −0.25); to date, the origins of their association remain unexplored.

Extant literature has also examined the association between anxiety and performance within specific contexts. One domain that has received extensive interest is mathematics. Mathematics anxiety, the negative feelings and emotional reactions elicited by mathematics or by the prospect of doing a task related to it^5^, varies in degrees of severity and is observed independently from levels of mathematical knowledge^6^. Studies have observed moderate negative correlations between mathematics anxiety and mathematics achievement across ages and educational curricula (average *r* = −0.30)^7^,^8^,^9^,^10^,^11^, with the exception of basic numerosity skills, which were found not to share an association with mathematics anxiety^12^.

Mathematics anxiety is also associated with lower rates of involvement in activities that require mathematics^7^, from taking any optional STEM (Science, Technology, Engineering and Mathematics) subject in school or university, to not choosing professional careers in the STEM fields^6^. This in turn is related with reduced opportunities to develop mathematical skills further^13^.

Similar cognitive mechanisms were found to characterise the association between anxiety and performance in domain-general contexts and in the domain of mathematics. One of the leading cognitive theories of anxiety, the attentional control theory (ATC)^14^ proposes that a disruption in working memory capacity is central to the negative link observed between general anxiety and performance. The framework suggests that high levels of anxiety interfere with working memory processes, leading to reduced performance efficiency and effectiveness. Several studies have supported the account^14^,^15^. Similarly, research has identified a disruption in working memory as characteristic of the association between anxiety and attainment in the domain of mathematics^16^,^17^,^18^.

As well as being characterised by similar underlying cognitive mechanisms in their association with performance, the two anxieties are associated with similar physiological indicators – including rapid pulse, nervous stomach, palpitations, dizziness, and tension headaches^19^,^20^. Recent studies, using neuroimaging and electrophysiological methods, have found an overlap in the brain areas associated with general and mathematics anxiety^17^,^21^. When children with high mathematics anxiety were presented with mathematical stimuli, they experienced increased activation and connectivity in the amygdala, which has also been associated with experiencing general anxiety, fear and negative emotions^17^. Another study using electro-encephalography (EEG) found that the same component (the error-related negativity –ERN)^22^ involved in error-monitoring behaviour in participants suffering from general anxiety^23^, was also implicated in error monitoring in mathematics anxiety^21^.

Although similarities between general and mathematics anxiety were observed in their physiological manifestations, cognitive and brain networks, their correlation is only moderate (average *r* = 0.35)^7^. This suggests that they may be separate constructs, manifesting themselves independently from one another, and characterised by different aetiologies.

Only one study to date has explored the aetiology of general and mathematics anxiety and of their association, in a sample of 12-year-old twins from the United States^24^. In this study, genetic factors contributed moderately to individual differences in general and mathematics anxiety. Individual-specific environmental factors explained the remaining variance in general and mathematics anxiety. Approximately 20% of the same genetic effects and 7% of the same nonshared environmental effects contributed to the origins of both general and mathematics anxiety. However, the majority of the aetiology was specific to each construct^24^. These findings suggest that, although the origins of general anxiety and mathematics anxiety partially overlap, their causes are also partly independent. However, the small sample size calls for caution when interpreting findings from this investigation.

Another context-specific anxiety construct that has received considerably less attention in the literature is spatial anxiety: the fear of performing tasks that have a spatial component^25^. Spatial anxiety has been linked to a decreased efficiency of orientation strategies^25^ and increased errors in a navigation task^26^. Spatial anxiety was found to emerge early on, with students in the early years of elementary school already showing variation in their degree of spatial anxiety^27^. In the same study, a negative association was observed between spatial anxiety and performance in a mental rotation task. Consistent with findings in the domain of mathematics anxiety^18^, this negative association was found predominantly in children with higher working memory skills. In fact, a similar disruption in working memory processes has been proposed to moderate the negative association between spatial anxiety and performance in spatial tasks^28^. Because spatial ability is a predictor of positive academic outcomes such as achievement in mathematics and science^28^,^29^, and success in STEM careers^30^, exploring the structure and origins of its affective correlates is of substantial importance.

To date, several aspects of spatial anxiety remain unexplored. Spatial anxiety has mostly been investigated in the context of navigation and orienting. Most of the existing self-report measures designed to assess spatial anxiety (e.g. the Way-Finding Strategy Scale^25^) have focused on exploring anxiety towards navigation or map reading skills. Only one instrument to date has been designed to assess anxiety in relation to other spatial abilities, such as mental rotation, visualization and object manipulation in young children (the Child Spatial Anxiety Questionnaire –CSAQ^27^). However, information on the factor structure of the CSAQ is not available, and only a total score for the questionnaire, combining items assessing several putative aspects of spatial anxiety, is recommended based on the internal validity of the measure (alpha = 0.56)^27^. Therefore, it remains unclear whether spatial anxiety is a unitary construct encompassing anxiety towards all spatial abilities (e.g. navigation, map reading, mental rotation, visualization, scanning etc.), or a multifactorial construct, characterized by several subcomponents. The aetiology of individual differences in spatial anxiety (or anxieties) also remains unexplored.

Up to now, only one study^31^ has explored the association between spatial anxiety and other anxiety constructs – including mathematics anxiety and general anxiety – finding only moderate correlations between them. However, their differentiation remains poorly understood. In fact, no study has investigated the potential overlap between measures of spatial, mathematics and general anxiety. Importantly, their association has not been explored within a genetically informative design. Behavioural genetics methodologies allow for the exploration of the origins of individual differences in specific traits as well as of the co-variation between multiple traits. Exploring the association between spatial, mathematics and general anxiety within a genetically informative design is likely to enhance our understanding of the origins of their association. This allows us to investigate to what extent the same genes, shared environments and individual-specific environments contribute to variation in anxiety across different domains. Importantly, applying a genetically informative design allows for the investigation of whether the domain-specificity of anxiety constructs, indicated by the moderate phenotypic correlations between measures, is reflected in their aetiology.

It is plausible that the aetiology of spatial anxiety is mostly independent from the other anxiety measures, as it was observed for mathematics and general anxiety^24^. This would support the view that anxiety is a complex multifactorial construct, comprising domain general and domain-specific aspects that are largely different in origins. On the other hand, as spatial and mathematical abilities correlate substantially phenotypically^31^,^32^, and have been found to share common neural correlates^33^ and genetic influences^34^, it is possible that the aetiology of spatial and mathematics anxiety also overlap substantially, above and beyond their relationship with general anxiety. Answering these questions related to the aetiology of spatial, mathematics, and general anxiety is likely to have important implications for both future researches (e.g. molecular genetic research aimed at identifying the specific genes related to anxiety in several domains) and practice (e.g. interventions).

Therefore, the present study has three main aims: (1) to explore the factor structure of spatial anxiety; (2) to investigate the origins of individual differences in spatial anxiety (or anxieties); and (3) to explore the association between general anxiety, mathematics anxiety and spatial anxiety using a genetically informative design, with the aim of addressing whether they are separate constructs phenotypically and aetiologically. Findings from this investigation are likely to have important implications for interventions aimed at alleviating anxiety in both general and specific contexts.

## Methods

### Participants

The sample included 2928 twins (1464 pairs): 586 monozygotic (MZ) and 878 dizygotic (DZ) pairs; 392 pairs were MZ females, 194 pairs were MZ males, 315 pairs were DZ same-sex females, 157 pairs were DZ same-sex males and 406 pairs were DZs of opposite sex. Participants were drawn from the Twins Early Development Study (TEDS), a large-scale multivariate longitudinal twin registry based in the United Kingdom. All families living in England and Wales who had twin-births between 1994 and 1996 were contacted by the office of National Statistics and asked to take part in the study. More than 16,000 families took part at first contact, and more than 10,000 twins are still contributing to the study. *Important, TEDS was and still is representative of the UK population*^35^. The TEDS sample comprises 4 birth cohorts, and not all cohorts participate in every study. The current study included participants recruited from the first two TEDS cohorts, aged between 18 and 21. The study was approved by the King’s College London ethics committee, and was conducted in accordance with the approved guidelines. Participants provided informed consent.

### Measures

#### General Anxiety

The 7-item Generalized Anxiety Disorder Scale (GAD-7)^36^ was used as a measure of anxiety. The GAD-7 was originally developed to assess generalized anxiety disorder in clinical samples^37^. Generalized anxiety disorder reflects distress caused by uncontrollable worry about potential future negative events^38^ As well as measuring generalized anxiety disorder, GAD-7 was found to be accurate in identifying other related conditions, part of the anxiety umbrella, including panic disorder, social anxiety and post-traumatic stress disorder^39^. GAD-7 has been validated and is considered a reliable measure of anxiety in the general population^36^. Evidence for the validity of GAD-7 as a measure of anxiety in the general population is shown by (1) its correlations with individual differences in traits that are usually associated with anxiety, such as depression (positively) and self-esteem (negatively) and (2) by the large differences in the GAD-7 mean scores between samples from the general population, primary care, and patients diagnosed with generalized anxiety disorder. The scale asks participants: ‘*How often in the past month have you been bothered by the following problems*?’. Participants have to rate the 7 items of the GAD-7 on a 4-point scale, from 1 = not at all to 4 = nearly every day. Examples of items are: ‘*Not being able to control worrying*’, ‘*Have trouble relaxing*’, and ‘*Feeling afraid as if something awful might happen*’. The self-report measure was administered online. The GAD-7 was previously found to be internally valid (*α* = 0.89) and reliable (test-retest correlation of 0.64)^36^. In our sample the GAD-7 was also found to be internally valid (*α* = 0.91).

#### Mathematics Anxiety

A modified version of the Abbreviated Math Anxiety Scale (AMAS)^40^ was administered to assess mathematics anxiety. The AMAS asks participants to rate how anxious they would feel when facing several mathematics-related activities. The measure includes 9 items that are rated on a 5-point scale ranging from ‘not nervous at all’ to ‘very nervous’. Examples of items are: ‘*Reading a maths book*’ and ‘*Listening to a maths lecture*’. We modified some of the existing items slightly in order to make the scale age appropriate for our sample, as all of our participants had left school, and some were no longer in education (please refer to the SOM for additional details on all the items included). The AMAS has been widely used and shows excellent internal validity (*α* = 0.90)^40^. Our modified version of the AMAS also showed excellent internal validity (*α* = 0.94) and showed good test-retest reliability (*r* = 0.85).

#### Spatial Anxiety

In order to assess several aspects of spatial anxiety we developed a 10-item questionnaire. Some of the items are loosely based on the Way-Finding Strategy Scale^25^, whereas other items were created for the purpose of the present investigation. Participants were asked to rate on a scale from 1 to 5 (1 = not at all, and 5 = very much) how anxious they would feel in situations involving spatial skills such as navigation, way-finding, mental rotation and spatial visualization. Exploratory factor analysis (see Supplementary Table S1) showed that the scale comprised two main factors: (A) a Navigation Anxiety factor and (B) a Rotation/Visualization Anxiety factor. The navigation anxiety factor included items such as: ‘*Finding your way around an intricate arrangement of streets*’, ‘*Trying a new shortcut without using a map*’, and ‘*Following somebody’s instructions to get somewhere*’. The factor showed very good internal validity (α = 0.86). The Rotation/Visualization anxiety factor included items such as ‘*Having to complete a complex jigsaw puzzle*’, and ‘*Having to rotate objects in your mind’*. This second factor also showed good internal validity (α = 0.78; see Supplementary Table S1 and Figure S1 for more details on the factor structure of spatial anxiety).

### Analyses

#### Phenotypic Analyses

We conducted Principal Component Analysis (PCA), to explore the factor structure of the newly developed spatial anxiety scale. We then conducted Confirmatory factor analysis (CA), using the statistical package MPlus^41^, to test whether the factor structure emerging from the exploratory PCA was the solution that best fitted the data. Once constructs had been identified and composite variables created, we explored their distribution associations using descriptive statistics and correlation. We also conducted univariate analyses of variance (ANOVA) to explore sex differences in all measures.

#### Genetic Analyses

##### The Univariate ACE Model

We applied the twin method, specifically the univariate ACE model, to investigate the origins of individual differences in anxiety measures. The twin method capitalises on the fact that monozygotic twins (MZ) share 100% of their genetic makeup and dizygotic twins (DZ) share on average 50% of the genes that differ between individuals, and on the assumption that both types of twins who are raised in the same family share their environments to approximately the same extent^42^. Comparing how similar MZ and DZ twins are for a given trait, it is possible to estimate the relative contribution of genes and environments to variation in that trait. The twin method decomposes the variance in a trait into additive genetic (A), shared environmental (C) and nonshared environmental (E) influences. Additive genetic factors are the sum of the effects of all alleles at all loci contributing to the variation in a trait or to the co-variation between traits. Shared environmental factors are environmental factors that contribute to similarities between family members. Nonshared environmental factors are those that do not contribute to similarities between family members. In the model, nonshared environmental variance also includes any measurement error^43^.

Genetic influence can be estimated by comparing intraclass correlations for MZ and DZ twins. A greater similarity between MZ twins than between DZ twins for a specific trait indicates a degree of genetic influence on the variance of that trait. Heritability, the amount of variance in a trait that can be attributed to genetic variance, can be calculated as double the difference between the MZ and DZ twin correlations. The univariate ACE model fitting applies full structural equation modelling to the estimation of heritability, shared environmental and non-shared environmental effects. Applying full structural equation modelling rather than comparing correlations, allows for the assessment of the goodness of fit of the model by comparing it to the saturated model (the model based on the observed data), and to more parsimonious models. Additionally, the univariate model estimates confidence intervals for all parameters^44^.

##### Full Sex Limitation Model

The univariate model can be extended to the full sex limitation model in order to explore whether the aetiology of individual differences in a trait differs depending on sex. The full sex limitation model allows for the investigation of both qualitative and quantitative sex differences. Qualitative sex differences are observed if different genetic and/or environmental factors influence of a given trait in males and females. On the other hand, quantitative sex differences are observed when the factors influencing the variation in a given trait are the same (i.e. same genes and same environments) for males and females, but the magnitude of their effects differs across sexes. The full sex limitation model is explained in more detail in the Supplementary Material (SOM) and elsewhere^45^.

##### Correlated Factors Model

The univariate model can be extended to multivariate models to investigate the origins of the correlation between traits. The correlated factors model (Figure S2) allows for the decomposition of the covariance between two traits into genetic, shared and non-shared environmental sources of variance, which are derived from the comparison of the cross-twin cross-trait correlations, obtained for MZ and DZ twin pairs. Cross-twin cross-trait correlations describe the association between two variables, with twin 1 score on variable 1 correlated with twin 2 score on variable 2. Cross-twin cross-trait correlations are calculated separately for MZ and DZ twins. A higher cross-twin cross-trait correlation for MZ than for DZ twins indicates that genetic factors have a degree of influence on the phenotypic relationship between the two traits. For example, the fact that the correlation between general anxiety for twin 1 and mathematics anxiety for twin 2 is higher for MZ than for DZ twins indicates a degree of genetic influence on the co-variance between general and mathematics anxiety. From the estimates obtained for each pairwise association, it is possible to derive the proportion of the phenotypic correlation between variables that can be attributed to genetic, shared and non-shared environmental influences^46^.

##### Independent Pathway Model

While the correlated factors model allows for the investigation of the aetiology of the co-variation between pairs of variables, multivariate models allow for the exploration of the common aetiology across multiple variables. For example, the independent pathway model^47^ (Fig. 2) allows for the investigation of the common aetiology between all variables entered in the model. The model decomposes the common variance between traits into: common and specific genetic (A), shared environmental (C) and nonshared environmental (E) influences. The magnitude of the genetic and environmental influences shared between all variables included in the model, is indicated by the size of the common A, C and E paths. This allows for the investigation of the extent to which the same genes and same environments are implicated in the origins of the co-variation between all traits included in the model. The effect of the residual (not shared between the variables included in the model) genetic, shared and non-shared environmental influences on every variable is indicated by the specific A, C, and E path estimates (see Figure S3).

## Results

### Factor Structure of Spatial Anxiety

To create a fully independent sample, all phenotypic analyses were conducted using data from one randomly-selected member of each twin pair. Similar results were obtained when the same analyses were performed on the other half of the sample – providing a built-in replication. We acknowledge, that this does not provide a full replication, as the other half of the sample did not provide us with the fully independent sample (i.e. including the other twin within each pair, who are genetically related).

PCA was used to explore the factor structure of anxiety. All the items included in the three anxiety measures (general anxiety, mathematics anxiety and spatial anxiety) were included in the analyses. Four clear factors emerged (Supplementary Figure S1 and Table S1). The first factor included all the items in the mathematics anxiety scale and explained 35.8% of the total variance. The second factor included all the items in the general anxiety scale and explained 13.2% of the total variance. The third factor, including six out of the ten items included in the spatial anxiety questionnaire, explained 9.3% of the variance; all items were relevant to navigation and way finding, therefore, we named this factor navigation anxiety. The fourth factor, explaining 6% of the variance, included three other items of the spatial anxiety scale; all describing the anxiety experienced while performing small-scale spatial tasks, such as mental rotation and visualization. This fourth factor was named rotation/visualization anxiety. Only one item in the spatial anxiety questionnaire loaded similarly on both factors 3 and 4, and was excluded from composite creation and further analyses.

Confirmatory factor analysis (FA) corroborated the factor structure observed from PCA. The four-factor model was the best fit for the data if compared to more parsimonious models (Supplementary Table S2).

### Descriptive Statistics and Correlations

Descriptive statistics for all anxiety measures are reported in Supplementary Table S3.

Pairwise associations between all variables are reported in Table 1. Correlations between all anxiety measures were moderate, with *r* coefficients ranging from 0.26 to 0.45.

### Sex differences

Table S4 presents the results of four ANOVAs, performed to explore sex differences in all anxiety measures. We observed significant sex differences for all measures, with females showing higher anxiety scores than males. However, sex only accounted for between 1.3% and 5.5% of the variance in anxiety. For subsequent analyses, the measures were corrected for the small age and sex differences using linear regression.

### Full Univariate Sex Limitation Models

Because we found significant, although small, phenotypic sex differences for all measures, we ran four univariate full sex limitation models (Supplementary Table S5) to investigate whether the aetiology of variation in anxiety measures was the same or different for males and females. We did not find qualitative sex differences, indicating that the same factors contributed to individual differences in all measures of anxiety for both males and females. The results indicated some significant quantitative sex differences in the aetiology of all measures; however, the confidence intervals around A, C ad E estimates for boys and girls were largely overlapping. Consequently, we included all MZ and DZ pairs in our analyses in order to maximise power. Although our sample included more than 1,400 twin pairs, we may have lacked power to detect small quantitative sex differences^48^.

### The Aetiology of Individual Differences in Anxieties

Univariate genetic analyses were used to explore the origins of individual differences in the four anxiety variables. Based on the observed intraclass correlations (Table 2), we ran four univariate ADE models to investigate the origins of individual differences in general, mathematics, navigation and rotation/visualization anxiety. The ADE model (described in SOM) decomposes the variance in a trait into additive genetic (A), non-additive genetic (D) and non-shared environmental (E) components. After comparing model fit indices (Supplementary Table S7), the more parsimonious AE model was found to be the best fit for all variables, indicating that non-additive genetic influences did not contributed significantly to explaining variation in anxiety measures.

Table 2 shows that additive genetic factors (A) contributed moderately to variation in all anxiety measures. Non-shared environmental factors (E), which include measurement error, accounted for the rest of the variance in all measures.

### The Origins of the Co-variation between Measures of Anxiety: Multivariate Genetic Analyses

**Figure 1:**
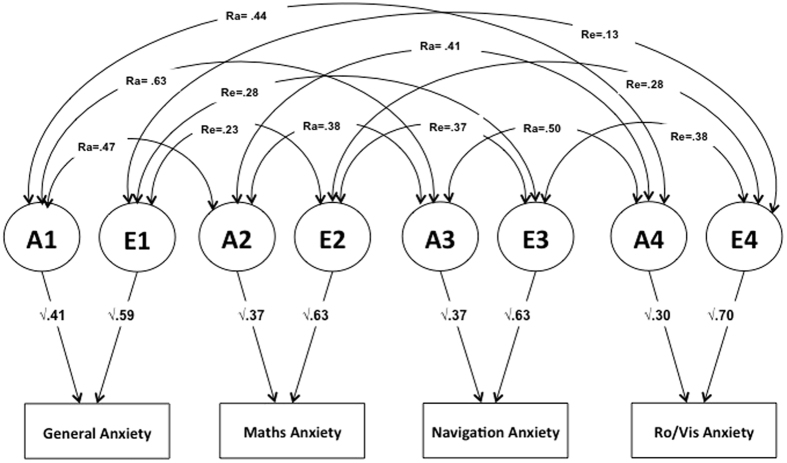
Correlated Factors Model for the association between general anxiety, mathematics anxiety, navigation anxiety and rotation and visualization anxiety. Ra = genetic correlation, Re = nonshared environmental correlation.

Figure 1 and Table 3 present the results of the correlated factors model. The more parsimonious AE model best fitted the data (Supplementary Table S8), indicating that shared environmental factors did not contribute to explaining the origins of the co-variation between measures of anxiety. Genetic correlations for all associations were moderate to strong, ranging from 0.38 to 0.63. Nonshared environmental correlations were weak to moderate, ranging from 0.13 to 0.38.

Genetic factors explained about half or more of the moderate correlations between anxiety variables (between 38% and 65%; Table 3). Non-shared environmental influences, which also encompass measurement error, explained between 35% and 62% of the phenotypic correlations between measures.

### Common Aetiology Across All Anxiety Measures: the Independent Pathway (IP) Model

In order to explore whether our data could be best summarised by a common genetic and environmental sources of variance across all measures, we ran an IP model. The model estimates the extents to which aetiological influences are common to several measures. The IP model also explores the aetiology of the variance that is not shared between variables.

**Figure 2:**
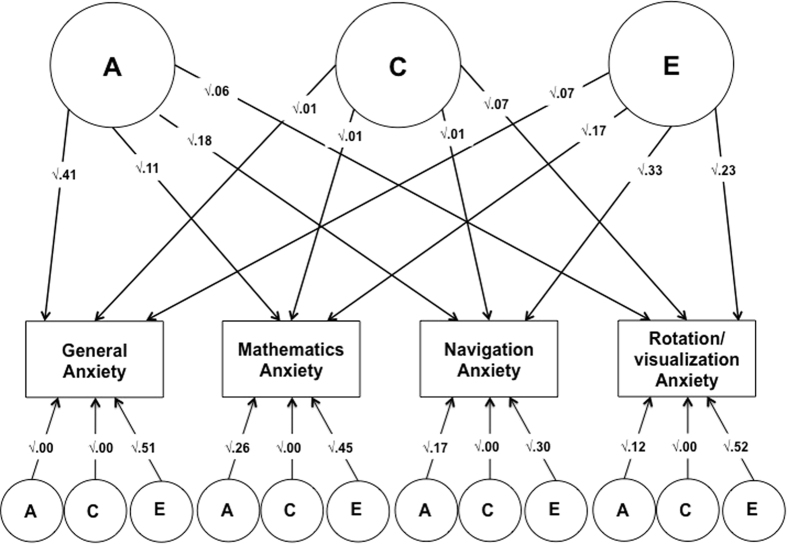
Independent Pathway Model looking at the origins of the association between general, mathematics, navigation and rotation/visualization anxiety. All paths are standardized and squared.

Figure 2 and Table 4 report the results of the independent pathway model. Table 4 presents the standardized paths estimates for the model. Figure 2 presents the standardized squared paths estimates. We found that, although some genetic and nonshared environmental influences were shared across the four anxiety measures, the aetiology of each anxiety construct was largely specific, as evidenced by the significant and substantial residual A and E estimates.

We subsequently ran a common pathway (CP) model (SOM), testing whether the aetiology of the four anxiety measures could be best described by one common latent factor encompassing genetic and environmental sources of influence. We found the CP model to be significantly lower in fit than the IP model, indicating that one latent factor encompassing all the common A, C and E influences could not best summarise the aetiology of the co-variation between the four anxiety measures (Supplementary Table S9).

## Discussion

The present study had three main aims: (1) to explore the factor structure of spatial anxiety; (2) to investigate the origins of individual differences in spatial anxiety; and (3) to explore the association between general, mathematics and spatial anxiety using a genetically informative design. We found that our measure of spatial anxiety included two distinct constructs: navigation anxiety –experienced in situations involving navigation and way-finding activities– and rotation/visualization anxiety –relevant to smaller-scale spatial activities such as mental rotation, visualization and object manipulation. Navigation and rotation/visualization anxiety were also largely independent from mathematics and general anxiety.

The factor structure of spatial anxiety, as well as the association between its components and mathematics anxiety and general anxiety, had not been previously investigated. The majority of previous research focused on exploring spatial anxiety only in the context of navigation and way-finding activities. Our results highlight the importance of considering another, largely separate, aspect of spatial anxiety, experienced when performing tasks such as mental rotation, visualization and object manipulation. This is consistent with studies that did not find an association between self-reported navigation ability and mental rotation^49^. These findings led to the speculation that navigation ability is mostly independent from smaller scale spatial abilities such as mental rotation^50^. Future investigations exploring the association between navigation anxiety, rotation/visualization anxiety and spatial abilities are needed in order to shed some light not only on the factor structure of spatial abilities, but also on the specificity of the association between anxiety and performance in the domain of spatial skills.

We found that females showed significantly higher levels of anxiety than males did in all domains. However, effect sizes were weak. Several previous investigations have reported sex differences in general and mathematics anxiety, usually finding that females experienced higher levels of anxiety^31^,^51^,^52^. Results are also consistent with a study that found that females experienced higher levels of way-finding anxiety than males^32^. Socio-cultural factors, such as the gender stereotype surrounding mathematics and, more generally, STEM subjects may contribute to these observed sex differences in anxiety. For example, women who value mathematics, and are acquainted with the social stereotype that women tend not to do as well as men in mathematics, tend to be the most sensitive to the pressure of gender stereotype and to feel anxious about mathematics^53^. Additionally, the higher levels of anxiety reported by females in every domain may partly depend on their greater willingness to disclose their levels of anxiety, if compared to males. This is consistent with findings showing that females reported higher trait mathematics anxiety; however, no sex differences were observed when state mathematics anxiety was measured straight after mathematics lessons^54^.

Little evidence was found for sex differences in the genetic and environmental architecture of anxiety, suggesting that the same factors are implicated in the aetiology of individual differences in anxiety to a similar extent in males and females.

All anxiety constructs were moderately heritable. Nonshared environmental factors, which are factors that do not contribute to similarities between twins raised in the same family, explained the remaining variance in all measures. Although it is reasonable to assume that shared environmental factors substantially influence anxiety levels, our study did not find any significant variance explained by these factors. This is consistent with previous research that found that shared environmental factors explained little or no variance in the aetiology of other non-cognitive traits related to individual differences in performance, such as motivation^55^ and personality^56^.

Our results are in line with those presented in the Wang *et al*. study in a younger sample of 12-year-old students^24^. As heritability estimates are specific to the population for which they are calculated at a particular time^57^, it was important to explore whether genetic factors played a similar role in explaining individual differences in a sample of older participants from the UK. Moreover, our study was the first to explore the origins of variation in spatial anxiety. Navigation anxiety was found to be moderately heritable, with genetic factors explaining 37% of individual differences in the trait. Rotation/visualization anxiety was found to be less heritable, with genetic factors explaining 30% of its variance.

Although all anxiety constructs constituted independent factors, all measures correlated moderately. We found that genetic factors explained about half or more of these phenotypic associations. For example, we found a strong genetic correlation between navigation and rotation/visualization anxiety, indicating that many of the same genes are implicated in individual differences in both measures. The strong genetic correlation between navigation and rotation/visualization anxiety explained nearly half of their moderate phenotypic correlation, and nonshared environmental factors explained the remaining portion. These findings are in line with previous research exploring the origins of the association between mathematics and spatial abilities. In fact, genetic influences were found to explain the largest portion of the covariance between mathematics and spatial abilities in a sample of 16 year-old TEDS twins^34^.

Due to the overlapping aetiologies between pairs of anxiety variables, we explored whether the same aetiological influences underlined all anxiety constructs. Our results showed that some genetic and nonshared environmental influences were common to all anxiety measures, indicating that some of the same genes and nonshared environments are implicated in individual differences in all anxiety constructs. However, significant specific genetic and non-shared environmental influences were also observed. The aetiological overlap between anxiety variables is consistent with research suggesting that partly the same physiological^19^, cognitive^6^ and brain^21^ processes are implicated in both general and mathematics anxiety. At the same time, the specificity observed in the aetiology of each measure is consistent with studies suggesting that mathematics and spatial anxiety manifest themselves independently from general anxiety^24^,^32^.

The specific cognitive and neural processes characterising mathematics and spatial anxiety remain mostly unexplored, as research looking into the brain correlates of mathematics anxiety has mainly focused on exploring the process shared with general anxiety. However, our results indicate a large degree of specificity in the aetiology of general, mathematics and spatial anxiety, which is likely to translate to specific neuronal and cognitive processes characterising these constructs. This is in line with evidence suggesting that mathematics anxiety is associated with a disruption in the subsystem of visual working memory, while general anxiety interferes with the verbal working memory system^58^. An interesting development for future research would be to identify the common and specific processes underlying different anxiety constructs, including specific genes and environments contributing to the development of domain-general and domain-specific anxiety constructs. The finding that a substantial degree of genetic specificity characterizes general, mathematics and spatial anxiety is useful for informing future quantitative and molecular research. For example, future genome-wide association studies (GWAS) should take into account these results, which suggest that multivariate GWAS (combining samples with data on academic anxiety and general anxiety to improve power) will have little hope for finding genetic variance common to all scales. Additionally, future quantitative research exploring the association between anxiety and achievement should take this domain-specificity into account.

To date, only one study has explored the origins of the association between mathematics anxiety and mathematics problem solving skills after accounting for general anxiety, finding a specific genetic link between mathematics anxiety and problem solving skills^24^. The specificity of the association between anxiety and performance remains unexplored in the spatial domain, as well as the specificity of the association between the subcomponents of spatial anxiety and different spatial abilities. It is possible that domain-specific anxieties would share a specific association with performance in that domain, above and beyond other anxiety measures. For example, it is possible that a specific association exists between navigation anxiety and navigation ability, above and beyond other domain-specific anxiety and ability constructs. Exploring the differential relationship between general, mathematics and spatial anxiety constructs and performance is part of our future plans. Moreover, the origins of these associations have not been investigated, and it is unclear whether specific genetic and environmental influences underlie the association between anxiety and performance in domain-specific contexts. We plan to explore these issues in our future research.

The present results also have important potential implications for interventions aimed at reducing anxiety, as they call for the need to intervene at the domain-specific level. For example, the majority of interventions aimed at alleviating the negative symptoms of mathematics anxiety have applied techniques that were found to be successful in diminishing general anxiety, with largely unsuccessful results^59^,^60^.Our findings suggest that interventions targeting general anxiety might only address a small part of the problem experienced by students suffering from mathematics and spatial anxiety and call for the need of considering the specificity of each anxiety construct.

The current study presents some of the limitations common to twin studies. One assumption of the twin method is the equal environments assumption, the idea that MZ and DZ twin pairs growing up in the same family share the same degree of environmental similarity. Although there is evidence suggesting that MZ twins are more likely to experience similar environments than DZ twins, for example being treated more similarly, studies have shown that sharing more environmental experiences did not impact on the degree of their phenotypic concordance^61^. A further limitation is that the twin method does not take into account genotype-environment effects such as assortative mating, genotype-environment correlation and gene-environment interaction. These limitations of the methodology are discussed in detail elsewhere^50^. Additionally, we only used self-reported measures of anxiety. Combining self-reports with other types of assessment, such as for example measuring physiological symptoms, skin conductance reactivity^62^, or cortisol levels^63^, would likely provide more in depth phenotypic information on all anxiety measures and the way they are manifested.

To conclude, the results of the present investigation support a multifactorial view of anxiety, both at the phenotypic and aetiological level. Our findings point to the importance of studying anxiety for specific domains. Although specific anxiety constructs show an association with the broader general anxiety domain, considering general anxiety alone is likely to provide only a partial picture of the apprehension experienced by individuals struggling with anxiety in specific fields. We found genetic factors to play a significant role in explaining variation in anxiety measures and their co-occurrence. Future genetic studies are likely to be able to identify the polygenic bases of anxiety constructs. Identifying the genetic bases of anxiety and of domain specific anxiety constructs is a priority, as the anxiety in many fields is negatively associated with emotional wellbeing as well as cognitive performance. The findings emerging for the current study benefit future research as well as practice, by providing useful knowledge for future studies and interventions aimed at reducing anxiety and at alleviating its negative association with performance.

## Additional Information

**How to cite this article**: Malanchini, M. *et al*. The genetic and environmental aetiology of spatial, mathematics and general anxiety. *Sci. Rep.* **7**, 42218; doi: 10.1038/srep42218 (2017).

**Publisher's note:** Springer Nature remains neutral with regard to jurisdictional claims in published maps and institutional affiliations.

## Supplementary Material

Supplementary Information

## Figures and Tables

**Table 1:** Correlations between anxiety measures.

	G anxiety	M anxiety	N anxiety	R/V anxiety
General anxiety	1	0.32**	0.44**	0.24**
Mathematics anxiety		1	0.41**	0.32**
Navigation anxiety			1	0.42**
Rotation/Vis anxiety				1

*Note: N* = 1464 (one twin per pair was randomly selected to control for non-independence of observation); **p < 0.001.

**Table 2:** Intraclass correlations, heritability and environmental estimates for all anxiety measures with 95% confidence intervals.

	rMZ	rDZ	A	D	E
Gen Anxiety	0.44**	0.17**	0.41 (0.34, 0.48)	—	0.59 (0.52, 0.64)
Maths Anxiety	0.43**	0.09**	0.37 (0.19, 0.45)	—	0.63 (0.62, 0.69)
Nav Anxiety	0.40**	0.14**	0.37 (0.29, 0.44)	—	0.63 (0.57, 0.70)
Rot/Vis Anxiety	0.35**	0.07**	0.30 (0.22, 0.36)	—	0.70 (0.63, 0.77)

Note: **p < 0.01; 95% confidence intervals in parentheses, A = additive genetic influences; D = non-additive genetic influences; C = shared environmental influences; E = nonshared environmental influences.

**Table 3:** Phenotypic (*r*_P_), genetic (*r*_A_) and non-shared environmental (*r*_E_) correlations for pairwise associations.

Pairs of variables	*r*_P_ (95% CI)	*r*_A_ (95% CI)	*r*_E_ (95% CI)
		*Percentage of r*_*P*_	*Percentage of r*_*P*_
G anxiety & M anxiety	0.32 (0.29–0.34)	0.47 (0.44–0.61)	0.23 (0.16–0.25)
		*58*%	*42*%
G anxiety & N anxiety	0.42 (0.39–0.43)	0.63 (0.55–0.90)	0.28 (0.21–0.34)
		*59*%	*41*%
G anxiety & R/V anxiety	0.24 (0.21–0.27)	0.44 (0.32–0.72)	0.13 (0.06–0.18)
		*65*%	*35*%
M anxiety & N anxiety	0.38 (0.35–40)	0.38 (0.20–0.52)	0.37 (0.30–0.41)
		*38*%	*62*%
M anxiety & R/V anxiety	0.32 (0.28–0.34)	0.41 (0.26–0.62)	0.28 (0.23–0.34)
		*43*%	*57*%
N anxiety & R/V anxiety	0.42 (0.41–0.44)	0.50 (0.32–0.69)	0.38 (0.32–0.43)
		*40*%	*60*%

*Note*: G anxiety = general anxiety; M anxiety = maths anxiety; N anxiety = navigation anxiety; R/V anxiety = rotation/visualization anxiety; 95% CI = 95% confidence intervals; *r*_A_ = genetic correlation; *r*_E_ = nonshared environmental correlation; *r*_P_ = phenotypic correlation.

**Table 4:** Standardized paths for the Independent Pathway Model (95% confidence intervals).

Common Paths			
AC1	AC2	AC3	AC4
0.64 (0.50, 0.69)	0.33 (0.24, 0.42)	0.43 (0.31, 0.51)	0.25 (0.08, 0.39)
**CC1**	**CC2**	**CC3**	**CC4**
−0.11 (−0.33, 0.14)	0.08 (−0.04, 0.21)	0.11 (−0.63, 0.21)	0.26 (0.09, 0.40)
**EC1**	**EC2**	**EC3**	**EC4**
0.26 (0.20, 0.29)	0.42 (0.34, 0.49)	0.57 (0.50, 0.65)	0.48 (0.41, 0.55)
**Specific Paths**			
**AS1**	**AS2**	**AS3**	**AS4**
−0.00 (−0.34, 0.34)	0.51 (0.43, 0.56)	0.42 (0.25, 0.48)	0.35 (0.12, 0.54)
**CS1**	**CS2**	**CS3**	**CS4**
0.00 (−0.24, 0.24)	0.00 (−0.21, 0.21)	0.00 (−0.28, 0.28)	0.00 (−0.23, 0.23)
**ES1**	**ES2**	**ES3**	**ES4**
0.71 (0.67, 0.75)	0.67 (0.62, 0.72)	0.55 (0.47, 0.61)	0.72 (0.67, 0.78)

Note: AC1, AC2, AC3, AC4 = Common genetic variance between all anxiety measures; CC1, CC2, CC3, CC4 = shared environmental variance common to al anxiety measures; EC1, EC2, EC3, EC4 = nonshared environmental variance common to al anxiety measures; AS1 = genetic variance specific to general anxiety that is not shared with the other anxiety measures; AS2 = genetic variance specific to mathematics anxiety that is not shared with the other anxiety variables; AS3 = genetic variance specific to navigation anxiety that is not shared with the other anxiety variables; AS4 = genetic variance specific to rotation/visualization anxiety that is not shared with the other anxiety variables; CS1, CS2, C3, CS4 = specific shared environmental variance; ES1, ES2, ES3, ES4 = specific nonshared environmental variance.
