# Supplementary material for: The genetic and environmental aetiology of spatial, mathematics and general anxiety
Source: Sci Rep. 2017 Feb 21;7:42218. doi: 10.1038/srep42218 (PMC5318949; doi:10.1038/srep42218)
Supplement: Supplementary Information [file srep42218-s1.pdf]

Supplementary Material

The genetic and environmental aetiology of spatial, mathematics and general anxiety

Margherita Malanchini[1,2], Kaili Rimfeld[3], Nicholas G. Shakeshaft[3], Maja Rodic[4], Kerry Schofield[3], Saskia Selzam[3], Philip S. Dale[5], Stephen A. Petrill[6], & Yulia Kovas[1,2,3]

Figures:

Figure S1. Scree plot illustrating the factor structure of the anxiety measures

Figure S2. Correlated factors model

Tables:

Table S1. Factor loadings for the four anxiety measures
Table S2. Model fit indices for confirmatory factor analysis (CFA)
Table S3: Descriptive Statistics for the four anxiety measures
Table S4: Phenotypic sex differences, univariate ANOVAs
Table S5. Twin correlations across sex and zygosity groups
Table S6. Univariate additive genetic (A), shared environmental (C) and nonshared environmental (E) estimates for males and females separately (95% confidence intervals).
Table S7. Model fit indices for all univariate models and nested models
Table S8. Model fit indices for the correlated factors model
Table S9. Model fit indices for Cholesky decomposition, independent pathway model and common pathway model

Method:

The ADE model

Table S1. Factor loadings for the four anxiety measures

	**1**	**2**	**3**	**4**
1. Finding your way around an intricate arrangement of streets	0.11	0.22	**0.76**	0.11
2. Directing somebody to a place of interest when standing in a windowless room	0.16	0.15	**0.64**	0.25
3. Locating a vehicle in a very large car park or garage	0.12	0.18	**0.57**	0.29
4. Having to complete a complex jigsaw puzzle	0.17	0.07	0.20	**0.61**
5. Finding your way around an unfamiliar place	0.17	0.19	**0.81**	0.04
6. Trying a new shortcut without using a map	0.16	0.10	**0.76**	0.14
7. Following somebody's instructions to get somewhere	0.16	0.17	**0.65**	0.24
8. Having to visualise a 3D object from a 2D drawing	0.12	0.09	0.21	**0.81**
9. Having to rotate objects in your mind	0.15	0.10	0.18	**0.79**
10. Finding a product in the local supermarket if the shelves have been rearranged	0.13	0.13	0.36	0.49
11. Using maths tables in the back of a maths text book	**0.62**	0.18	0.10	0.30
12. Thinking about an upcoming maths test	**0.78**	0.15	0.26	-0.08

13. Watching teacher working out an algebraic equation	**0.80**	0.13	0.06	0.24
14. Taking an exam in a maths course	**0.80**	0.11	0.25	-0.13
15. Being given an assignment of difficult maths problems	**0.86**	0.10	0.21	0.04
16. Listening to a maths lecture	**0.82**	0.14	0.05	0.25
17. Listening to someone explaining a maths formula	**0.83**	0.16	0.07	0.26
18. Being given a surprise quiz	**0.80**	0.10	0.24	-0.01
19. Reading a maths book	**0.78**	0.12	0.01	0.29
20. Feeling nervous anxious or on edge	0.15	**0.77**	0.25	0.01
21. Cannot stop or control worrying	0.16	**0.84**	0.20	0.06
22. Worrying too much about different things	0.19	**0.82**	0.21	0.03
23. Having trouble relaxing	0.10	**0.83**	0.14	0.07
24. Being so restless it is hard to sit still	0.07	**0.72**	0.07	0.14
25. Becoming easily annoyed or irritable	0.09	**0.67**	0.07	0.08
26. Feeling afraid as something awful might happen	0.15	**0.75**	0.15	0.13

Note: method of rotation: Varimax; *N* = 1464, one twin out of each pair was selected to control for non-independence of observation; Factor 1 = mathematics anxiety; Factor 2 = general anxiety; Factor 3 = navigation anxiety; Factor 4 = rotation/visualization anxiety.

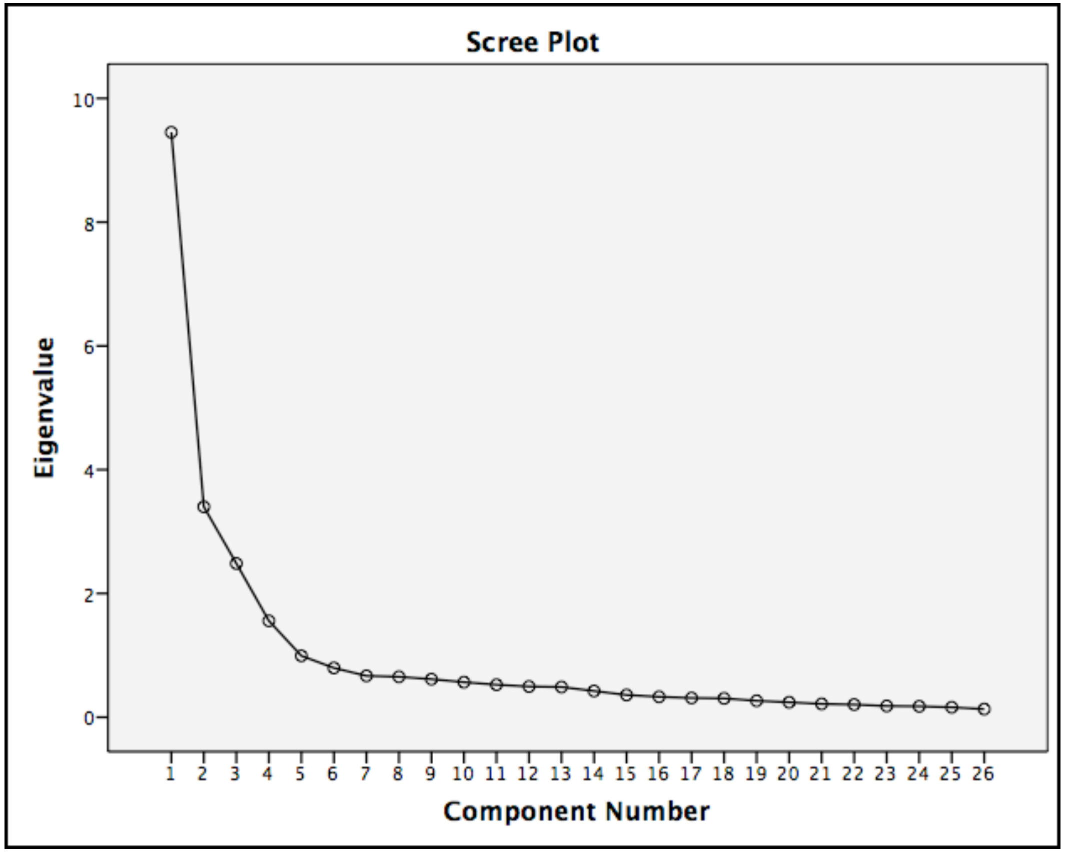

Figure S1. Scree plot illustrating the factor structure of anxiety measures.

Table S2. Model fit indices for Confirmatory Factor Analysis

	AIC	BIC	RMSEA	CFI	TLI	SRMR
**4 factors**	**87988.78**	**88419.74**	**0.08**	**0.88**	**0.86**	**0.05**
3 factors	93012.99	93443.95	0.10	0.82	0.80	0.06
2 factors	96481.61	96901.93	0.13	0.69	0.66	0.12
1 factor	101577.17	101992.17	0.17	0.49	0.45	0.15

Note: AIC = Akaike Information Criterion; BIC = Bayesian Information Criterion; RMSEA = Root Mean Square Error of Approximation; CFI = Comparative Fit Index; TLI = Tucker-Lewis Index, SRMR = Standardized Root Mean Square Residuals.

Table S3: Descriptive statistics

	General anxiety	Mathematics anxiety	Navigation anxiety	Rotation/Visualization anxiety
N	1511	1511	1511	1511
Mean	1.97	2.30	2.29	1.64
Std. Deviation	0.74	1.01	0.82	0.77
Skewness	0.87	0.72	0.64	1.37
Kurtosis	0.12	-0.28	0.06	1.69
Minimum	1.00	1.00	1.00	1.00
Maximum	4.00	5.00	5.00	5.00

Note: *N* = one twin out of each pair randomly selected to control for non-independence of observation.

Table S4. Univariate analyses of variance (ANOVA) examining sex differences in all variables

	Female *M (SD), N*	*Male* *M (SD), N*	*F*	*Partial η2*
General Anxiety	2.07 (.77) *N* = 965	1.78 (.64) *N* = 546	58.71**	0.037
Mathematics Anxiety	2.45 (1.03) *N* = 965	2.03 (.88) *N* = 546	64.95**	0.041
Navigation Anxiety	2.43 (.83) *N* = 965	2.02 (.72) *N* = 546	88.27**	0.055
Rotation/Visualization Anxiety	1.70 (.78) *N* = 965	1.52 (.72) *N* = 546	20.38**	0.013

Note: One twin per pair was randomly selected to control for non-independence of observation; **= $p < .001$

The aetiology of individual differences in anxiety constructs –sex by genotype interaction: the Univariate Sex Limitation model

Sex differences at the aetiological level are suggested when the correlation between opposite sex DZ pairs is significantly lower than the correlation between same sex DZ pairs. Table 4 reports the twin correlations for MZ and DZ twins depending on sex. The difference in the strength of correlations between DZ same sex and DZ opposite sex pairs suggests quantitative sex differences in the aetiology of individual differences in the variables.

No qualitative sex differences were found for any of the four anxiety measures. However, quantitative sex differences were detected in the aetiology of individual differences in all anxiety measures. Table S5 reports the estimates for additive genetic, shared environmental and nonshared environmental influences for each anxiety measure for boys and girls separately. Sex limitation models fitting suggested differences in the estimates of the contribution of genetic and environmental factors to variation in all anxiety measures for males and females ($p < .01$). However, the confidence intervals around the estimates for males and females were largely overlapping. The overlap in confidence interval suggests two things: (1) our analysis could not differentiate between genetic and environmental estimates for boys and girls with adequate power; and (2) the estimates for boys and girls were comparable. Consequently, we included all MZ and DZ pairs, including opposite sex twin pairs, in our subsequent analyses.

Table S5. Twin correlations across sex and zygosity groups.

	***r*MZm**	***r*MZf**	***r*DZm**	***r*DZf**	***r*DZos**
General anxiety	.51**	.42**	.24**	.22**	-0.01
Maths anxiety	.30**	.45**	.30**	0.05	0.01
Navigation anxiety	.42**	.40**	.20*	.14*	-.00
Rot/Vis anxiety	.30**	.33**	.31**	0.01	.00
N	194	392	157	315	406

Note: *r*MZm = correlation between monozygotic males; *r*MZf = correlation between monozygotic females; *r*DZm = correlation between dizygotic males; *r*DZf = correlation between dizygotic females; rDZos = correlation between dizygotic opposite sex twins; N = number of twin pairs in each group; ** = p< .01; * = p< .05.

Table S6. Univariate additive genetic (A), shared environmental (C) and nonshared environmental (E) estimates for males and females separately (95% confidence intervals).

	Am	Cm	Em	Af	Cf	Ef
G anxiety	.27 (.00, .51)	.23 (.03, .49)	.50 (.40, .61)	.32 (.10, .46)	.08(00, .26)	.59 (.51, .68)
M anxiety	.06(.00, .33)	.35 (.18, .45)	.65 (.55, .77)	.41 (.32, 48)	.02(.00, .20)	.59 (.51, .67)
N anxiety	.02 (.00, .33)	.37 (.11, .48)	.61 (.49, .72)	.40 (.23, .49)	.01 (.00, .14)	.59 (.51, .67)
R/V anxiety	.00 (.00, .20)	.30 (.12, .40)	.70 (.60, .81)	.28 (.18, .37)	.00 (.00, .18)	.71 (.63, .80)

Note: Am = estimate of genetic effects for males; Cm = estimates of shared environmental effects for males; Em = estimates of nonshared environmental effects for males; Af = estimate of genetic effects for females; Cf = estimates of shared environmental effects for females; Ef = estimates of nonshared environmental effects for females.

The aetiology of individual differences in anxiety constructs: The ADE model

The univariate model estimates the proportion of variance that is attributable to additive genetic (A), non-additive genetic (D), shared environmental (C), and non-shared (E) environmental influences by comparing the intraclass correlations for MZ and DZ twins for the trait of interest. Non-additive genetic effects (D) describe interactions between alleles at the same locus (dominance) and at different loci (epistatsis). The classic twin design, comparing MZ and DZ twins, does not allow to estimate all four sources of influence (A, D, C and E) within one model, as it only includes two coefficients of relatedness. Therefore, with the classic twin design it is possible to partition the variance into three sources of influences: A, E, and either C or D. The decision of including C or D into the model depends on the comparison of the correlations between MZ pairs and DZ pairs for the same trait (intraclass correlations). If the intraclass correlation for MZ twins is less than double that of DZ twins, shared environment is likely to have an influence on the trait. Consequently, C would be included in the model – ACE model. On the other hand, if the intraclass correlation for MZ pairs more than doubles that of DZ pairs, non-additive genetic effects are likely to play a role, and therefore D would be included in the model – ADE model.

Table S7. Model fit indices for all univariate models and nested models

	Baseline	Comparison	-2LL	df	AIC	*p*
(a) General Anxiety						
1	Saturated	-	7458.778	2675	2108.778	-
2	Saturated	ADE	7462.354	2681	2100.354	0.73
**3**	**ADE**	**AE**	7463.351	2682	2099.351	0.32
4	ACE	E	7594.550	2683	2228.550	0.00
(b) Mathematics Anxiety						
1	Saturated	-	7488.693	2675	2138.693	-
2	Saturated	ADE	7492.368	2681	2130.368	0.72
**3**	**ADE**	**AE**	**7501.925**	**2682**	**2137.925**	**0.03**
4	ADE	E	7594.550	2683	2228.550	0.00
(c) Navigation Anxiety						
1	Saturated	-	7494.874	2675	2144.874	-
2	Saturated	ADE	7497.674	2681	2135.674	0.83
**3**	**ADE**	**AE**	**7499.062**	**2682**	**2135.062**	**0.24**
5	ADE	E	7594.550	2683	2228.550	0.000
(d) Rotation/Visualization Anxiety						
1	Saturated	-	7517.452	2675	2167.452	-
2	Saturated	ADE	7526.233	2681	2164.233	0.19
**3**	**ADE**	**AE**	**7531.338**	**2682**	**2167.338**	**0.04**
4	ADE	E	7594.550	2683	2228.550	0.00

Note: -2L = negative 2 times log likelihood; df = degrees of freedom; AIC = Akaike Information Criterion

Table S8. Model fit indices for the Correlated Factors Model.

Baseline model	comparison	ep	- 2LL	df	AIC	*p*
Saturated	-	88	18615.906	8376	1863.9056	-
Saturated	Full ACE	34	18702.761	8430	1842.7607	0.003
Saturated	AE Model	24	18704.321	8440	1824.3212	0.023
**Full ACE**	**AE Model**	**24**	**18704.321**	**8440**	**1824.3212**	**0.999**
Full ACE	CE Model	24	18759.567	8440	1879.5669	0.000
Full AE	E Model	14	19085.388	8450	2185.3879	0.000

Note: -2L = negative 2 times log likelihood; df = degrees of freedom; AIC = Akaike Information Criterion.

Table S9. Model fit indices for Cholesky Decomposition, Independent Pathway Model and Common Pathway Model.

Baseline	Comparison	ep	-2LL	df	AIC	*p*
Saturated	-	88	28310.89	10652	7006.897	-
Saturated	Cholesky ACE	34	28388.47	10706	6976.469	.014
Cholesky ACE	Independent Pathway	28	28407.37	10712	6983.369	.004
Cholesky ACE	Common Pathway	23	28439.44	10718	7003.436	.000
Indep. Pathway	Common Pathway	23	28439.44	10718	7003.436	.000

Note: ep = number of parameters estimated by the model; -2LL = negative log likelihood, df = degrees of freedom; AIC = Akaike Information Criterion

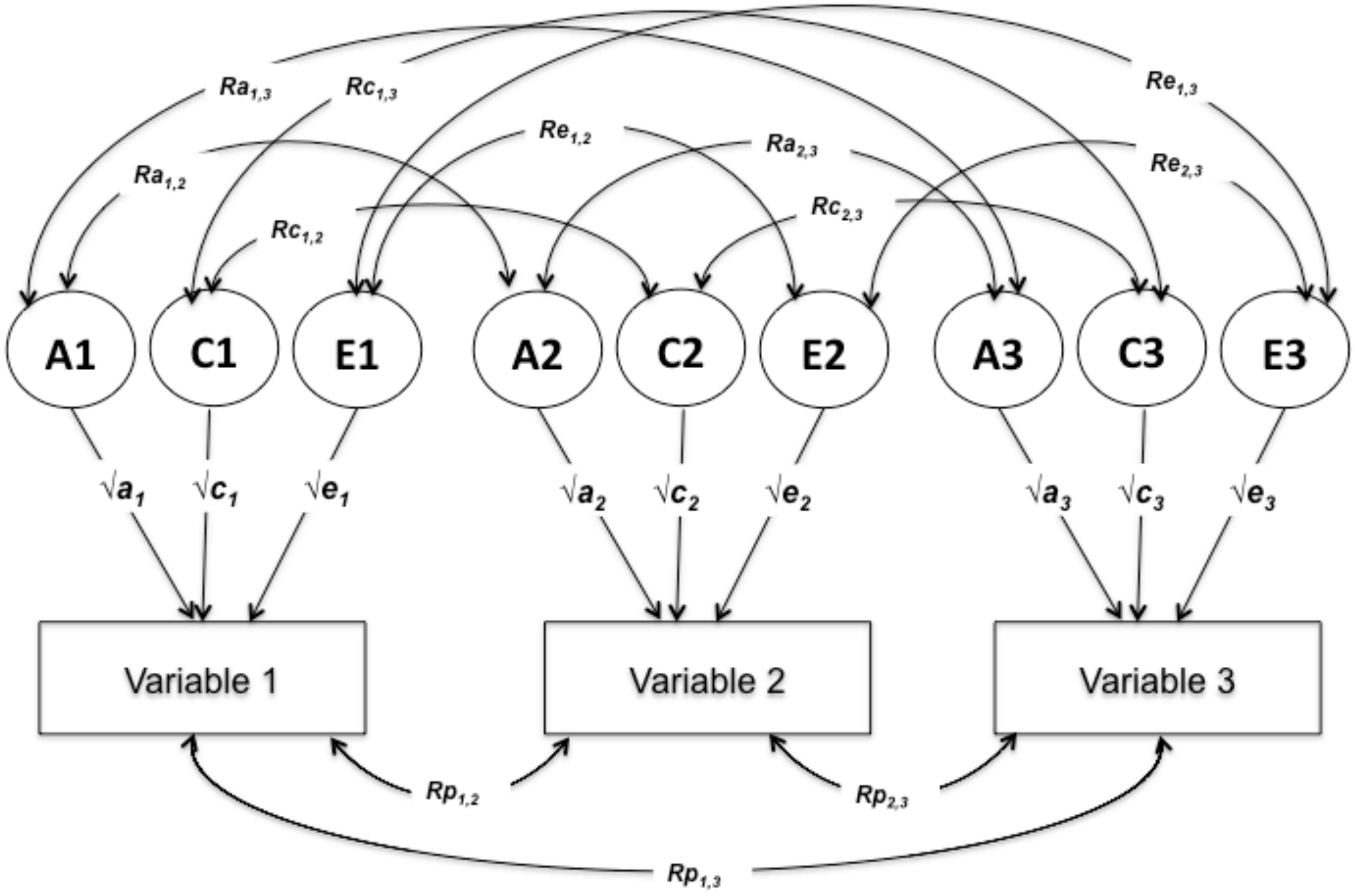

Figure S2. The correlated factors model; A = additive genetics; C = shared environment; E = non-shared environment; *Ra* = genetic correlation; *Rc* = shared environmental correlation; *Re* = non-shared environmental correlation; *Rp* = phenotypic correlation.

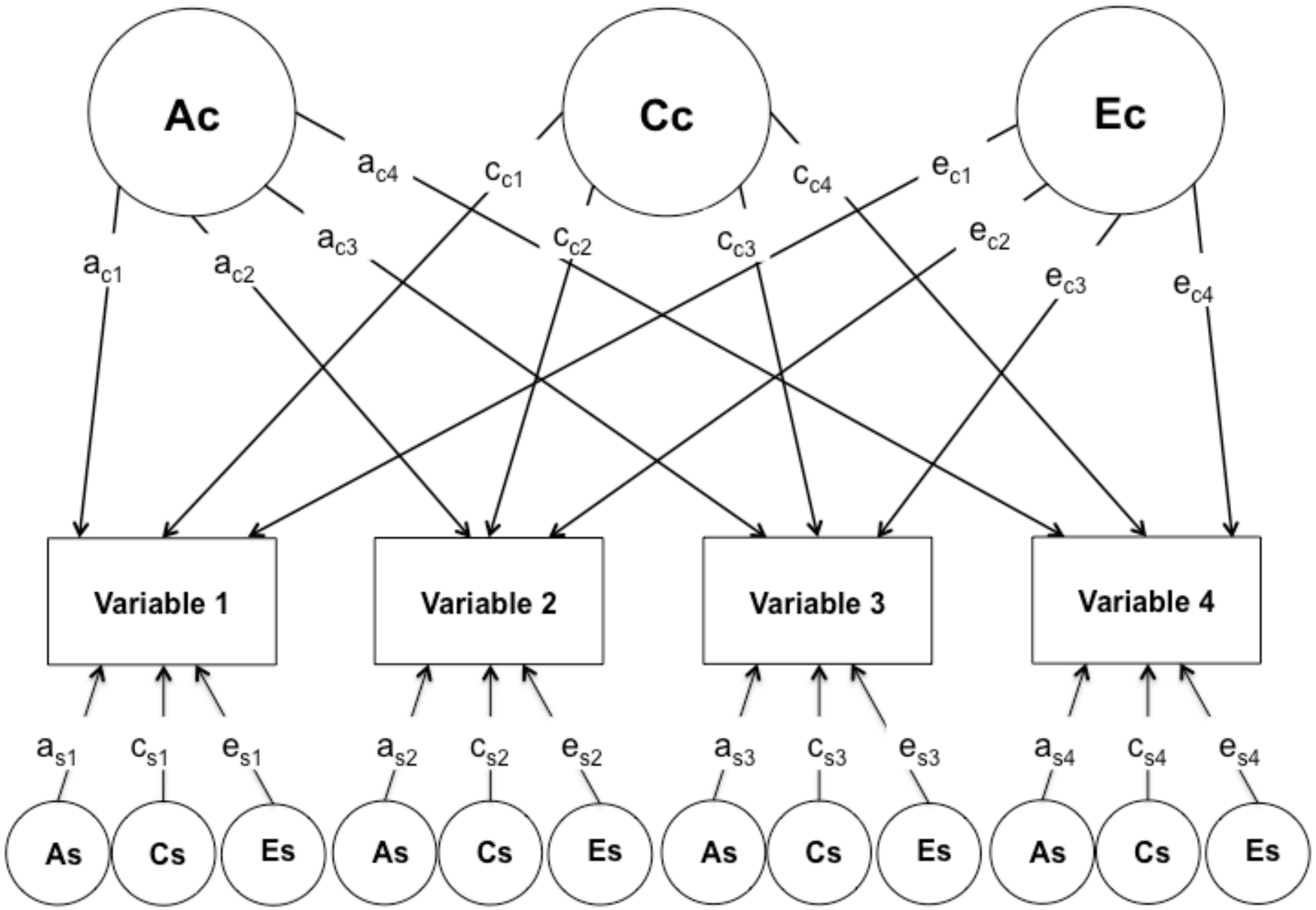

Figure S3. Independent pathway model; Ac = common A variance; Cc = common C variance; Ec = common E variance; As = specific A variance; Cs = specific C variance; Es = specific E variance.
